# Malaria in a vulnerable population living in quilombo remnant communities in the Brazilian Amazon: a cross-sectional study from 2005-2020

**DOI:** 10.1590/S1678-9946202466025

**Published:** 2024-04-19

**Authors:** Beatriz Costa Ribeiro, Carla Gisele R Garcia, Lilian Jéssica Passos Lima, João F. Guerreiro, Marinete Marins Póvoa, Maristela G. Cunha

**Affiliations:** 1 Universidade Federal do Pará, Instituto de Ciências Biológicas, Laboratório de Microbiologia e Imunologia, Belém, Pará, Brazil; 2 Secretaria de Saúde do Estado do Pará, Belém, Pará, Brazil; 3 Universidade Federal do Pará, Laboratório de Genética Humana e Médica, Belém, Pará, Brazil; 4 Instituto Evandro Chagas, Ananindeua, Pará, Brazil

**Keywords:** Malaria, Quilombo remnant communities, Para State, Amazon region, Brazil

## Abstract

Quilombo remnant communities are areas officially recognized by the Brazilian government as historical communities founded by formerly enslaved individuals. These communities are mostly located in the endemic areas of malaria in the Brazilian Amazon. We retrospectively described the prevalence of malaria among individuals living in 32 recognized quilombo remnant communities in the Baiao and Oriximina municipalities located in the Para State. The number of malaria cases and the Annual Parasitic Incidence (API) recorded by the Brazilian malaria surveillance system (SIVEP-Malaria) from January 2005 to December 2020 were analyzed. We found that all communities registered at least one case over the 16-year period, the most frequent parasitic species being *Plasmodium vivax* (76.1%). During this period, 0.44% (4,470/1,008,714) of the malaria cases registered in Para State were reported in these quilombo remnant communities, with frequencies of 10.9% (856/7,859) in Baiao municipality and 39.1% (3,614/9,238) in Oriximina municipality, showing that individuals living in these rural communities are exposed to malaria. These data indicate that effective surveillance requires improved measures to identify malaria transmission among vulnerable populations living in quilombo remnant communities in the Brazilian Amazon.

## INTRODUCTION

Malaria is an infectious disease caused by protozoa of the genus *Plasmodium* and is transmitted via the bite of female mosquitoes of the *Anopheles* genus^1-3^. In total, five species of parasites are recognized as etiological agents of human malaria, the most prevalent being *Plasmodium falciparum* and *Plasmodium vivax* ^2,4^. In 2022, a total of 151,530 malaria cases were registered in Brazil^4^. Malaria is a severe public health issue in the Amazon region, where 99% of all cases are recorded, 85% of which were caused by *P. vivax* ^4-7^.

Malaria parasites can infect any human population living in a transmission area. Among the nine Brazilian states belonging to the Amazon region, Para State has been considered a major endemic area for malaria over the last three decades, as it significantly contributes to malaria incidence and morbidity rates^5-7^. Therefore, it is important to consider the human populations living in rural localities of Para State, some of which are recognized as Quilombos^8-10^. The origin of quilombo communities dates back to the colonial period in Brazil, when enslaved African individuals who had fled from captivity in the 17^th^–18^th^ century founded villages, many of which on river banks^8^. Many well-established and federally recognized quilombo remnant communities currently exist along the Tocantins and Trombetas rivers, which cross the Baiao and Oriximina municipalities^10^.

The remaining communities of quilombos are ethnic groups according to self-attribution criteria. Anthropology defines them as organizational types founded by many slaves living in forests and organized in independent communities, each presenting characteristics for federal recognition, definition, and registration of their lands^11^. The subsistence strategies of these populations stemmed mainly from agricultural and extractive activities, in which high rates of human-mosquito contact occur^12,13^. Therefore, residents of these areas are highly exposed to the risk of malaria infection, a public health issue that has long affected human populations living in the Amazon region^5,7,14^. However, data on malaria epidemiology in these populations are extremely scarce, especially for quilombo remnant communities.

We performed a retrospective description and risk classification according to the annual parasitic incidence (API), used to classify malaria-endemic areas. This study can contribute to the implementation of effective measures to reduce the prevalence of malaria in risk areas, such as quilombo remnant communities in Para State, Brazil. In this study, we analyzed the prevalence of malaria over a 16-year period among people living in quilombo remnant communities based on data collected from the Brazilian malaria surveillance system (SIVEP-Malaria).

## MATERIALS AND METHODS

### Study design, study area, ethical aspects, and data collection

This is a retrospective description based on data from a cross-sectional study conducted in 2005–2020 on the number of malaria cases and risk of malaria transmission according to the API index. Thus, descriptive analysis was conducted to characterize the incidence of malaria in areas of quilombo remnant communities belonging to Baiao and Oriximina municipalities, Para State, Amazon. The localities were selected based on both official recognition by the Brazilian government^10^ and inclusion in the SIVEP-Malaria, with the identification of each community. No population or social characteristic were included when classifying one locality as a quilombo community.

**Figure 1:**
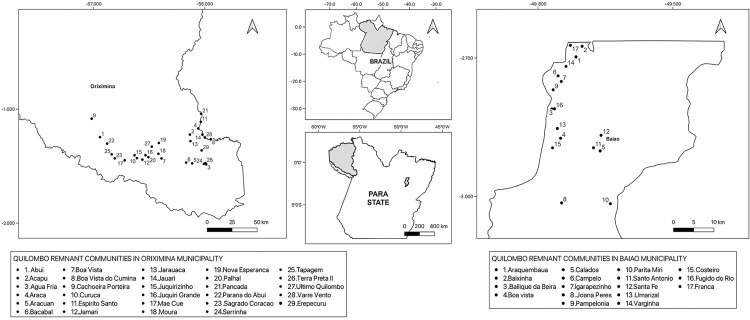
Map of study areas located in two municipalities of Brazil, Para State, Oriximina municipality (left) and Baiao municipality (right). Quilombo remnant communities in Baiao municipality (n=17), 14 officially recognized, and with data informed in the SIVEP-Malaria system: Araquembaua, Baixinha, Bailique da Beira, Boa Vista, Calados, Campelo, Igarapezinho, Joana Peres, Pampelonia, Parita Miri, Santo Antonio, Santa Fe, Umarizal, and Varginha; and three officially recognized, but without records in the SIVEP-Malaria system: Costeiro, Fugido do Rio, and Franca. Quilombo remnant communities in Oriximina municipality (n=29), 18 officially recognized, with data informed in the SIVEP-Malaria system: Agua Fria, Araca, Aracuan, Bacabal, Boa Vista, Cachoeira Porteira, Curuca, Espirito Santo, Jamari, Jarauaca, Jauari, Juquirizinho, Moura, Nova Esperanca, Parana do Abui, Serrinha, Tapagem, and Terra Preta II; and 11 officially recognized, but without records in the SIVEP-Malaria system: Abui, Acapu, Boa Vista do Cumina, Erepecuru, Juquiri Grande, Mae Cue, Palhal, Pancada, Sagrado Coracao, Ultimo Quilombo, and Varre Vento.

The study area comprised 32 quilombo remnant communities in Para State, Northern Brazil; 14 in the Baiao municipality; and 18 in the Oriximina municipality. These communities are officially recognized by the Brazilian government as historical communities founded by formerly enslaved individuals^9,10^, and their data on malaria are available. The Baiao and Oriximina municipalities are geographically very far apart, but there are many quilombo remnant communities in both regions, considering all municipalities of Para State (Figure 1). In Oriximina municipality, these communities are located in remote rainforest areas^11,15^.

Baiao municipality is located in the northeastern region of Para State, approximately 200 km from Belem city, capital of Para State, and is bordered by five municipalities: Mocajuba and Moju municipalities to the north and east, Tucurui and Breu Branco municipalities to the south, and Oeiras do Para municipality to the west. According to the 2022 census^16^, Baiao has 51,641 inhabitants in urban and rural areas, including 17 rural quilombo communities. In total, 14 of these communities are officially recognized and present data on malaria, available in the SIVEP-Malaria system. These communities include Araquembaua, Baixinha, Bailique da Beira, Boa Vista, Calados, Campelo, Igarapezinho, Joana Peres, Pampelonia, Parita Miri, Santo Antonio, Santa Fe, Umarizal, and Varginha. Costeiro, Fugido do Rio, and Franca municipalities are officially recognized but data in the SIVEP-Malaria system are unavailable.

Oriximina municipality is located in western Para State and is bordered by the Terra Santa, Faro, Juriti, and Obidos municipalities to the South and East; Amazonas and Roraima States to the West; and Guiana and Suriname to the North. According to the 2022 census^16^, Oriximina municipality accounts for 68,294 inhabitants in urban and rural areas, with more than half of this population living in rural areas, including 29 quilombo communities. Of these, 18 are federally recognized and present malaria data in the SIVEP-Malaria system, including Agua Fria, Araca, Aracuan, Bacabal, Boa Vista, Cachoeira Porteira, Curuca, Espirito Santo, Jamari, Jarauaca, Jauari, Juquirizinho, Moura, Nova Esperanca, Parana do Abui, Serrinha, Tapagem, and Terra Preta II. The other 11 communities, Abui, Acapu, Boa Vista do Cumina, Jarauaca-Erepecuru, Juquiri Grande, Mae Cue, Palhal, Pancada, Sagrado Coracao, Ultimo Quilombo, and Varre Vento, despite being officially recognized, lack malaria data in the SIVEP-Malaria system.

This study was approved by the Universidade Federal do Para Research Ethics Committee (CAAE Nº 55270822.1.0000.0018). Malaria data from January 2005 to December 2020 were collected from the SIVEP-Malaria system, Brazilian Ministry of Health, by an authorized employee of Para State Department of Health (SESPA), then analyzed in this study. These data are also available in epidemiological bulletins from the Brazilian Ministry of Health^17^.

A malaria case in Brazil is defined as an individual tested positive for *Plasmodium* spp. detection by microscopy. The data were collected from the SIVEP-Malaria system by place of infection when registered in the quilombo community officially recognized by Brazilian Government and data were analyzed by year, number of malaria cases, and annual parasitic incidence (API). The API was obtained directly from the SIVEP-Malaria system that used population baselines from the 2010 population census performed by Brazilian Institute of Geography and Statistics (IBGE).

The API index is a widely used indicator for monitoring malaria transmission in Brazil and for estimating the risk of infection within a given population over a certain period. API was estimated as follows: API = (NPT/TRP) *1,000, where NPT represents the number of positive tests in a year and TRP the total resident population in that year, multiplied by 1,000 at risk, expressing the number of malaria cases per 1,000 inhabitants in a given geographical area for the selected year^17,18^. The degree of risk of API is expressed as very low risk (< 1 case), low risk (< 10 cases), medium risk (10 – 50 cases), or high risk (> 50 cases)^18^.

### Statistical analysis

Descriptive analyses were performed via central tendency and dispersion measurements. The mean-standard deviation, as well as maximum and minimum values were obtained for the number of malaria cases and API for all communities.

## RESULTS

This study analyzed the occurrence of malaria cases over a 16-year period in 32 quilombo remnant communities in Para State. Both municipalities contain a high number of quilombo communities. However, only a few communities are officially recognized by the Government (Figure 1). The data recorded in the SIVEP-Malaria system showed a total of 4,470 notified malaria cases (Table 1). Of these, 856 and 3,614 cases were reported in the quilombo remnant communities in the Baiao and Oriximina municipalities, respectively. When compared to data derived from the entirety of Para State, 0.44% (4,470/1,008,714) of cases occurred in quilombola communities. Considering the total malaria cases in each municipality, however, the frequency of cases among quilombo communities was 10.9% (856/7,859) in Baiao and 39.1% (3,614/9,238) in Oriximina.

**Table 1:** - Malaria cases and Annual Parasitic Incidence (API) over 16 years (2005 – 2020) in endemic areas of the Para State and in quilombo remnant communities of Baiao and Oriximina municipalities.

Malaria endemic areas and communities	Malaria cases by locality of infection				Annual Parasitic Incidence (API)	

	Total	Average± SD*	range	%**	mean ± SD	range
Para State	1,008,714	63.0 ± 43.3	9,584 – 136,466	100	8.4 ± 6.1	1.2 – 18.7
Quilombo remnant communities in Baiao municipality	856	53.5 ± 68.4	0 – 246	0.08	12.5 ± 29.9	0 – 174.7
Baiao municipality	7,859	205.7 ± 200.0	8 – 3,203	0.78	19.3 ± 37.8	0.2 – 148.7
Quilombo remnant communities in Oriximina municipality	3,614	225.88 ± 440.9	0 – 1,779	0.36	84.9 ± 279.0	0 – 2,041.4
Oriximina municipality	9,238	577.3 ± 764.0	6 – 2, 687	0.91	9.8 ±13.3	0.1 – 45.8

*SD = Standard deviation. **% = comparison with the entirety of Para State.

We observed variations in the number of cases and API for the two study areas. Based on API means in quilombo remnant communities, variation was higher in Oriximina municipality (84.9 ± 279.0) than in Baiao municipality (12.5 ± 29.9). Nonetheless, we found no continuous malaria transmission at any locality, which indicates unstable and focal transmission. Another 224 cases were recorded in communities that have not yet been officially recognized, one in Oriximina municipality (Ariramba community) and six in Baiao municipality (Engenho, Florestao, Taperucu, Pocao, Cardoso, and Tambai-acu communities).

The distribution over time showed that the frequency of cases per year also differed between the studied areas (Table 2). In Baiao municipality, the year with the highest malaria incidence was 2018, with a total of 246 recorded cases, 66 of these in the same community. For Oriximina municipality, 2009 presented the largest number of recorded cases, with a total of 1,779, 543 of which reported in the same community. For the 16-year period, API varied among communities, from zero to 189 (Baiao municipality) and from zero to 2,041.4 (Oriximina municipality).

**Table 2:** - Malaria cases and Annual Parasitic Incidence (API) by year in 32 quilombo remnant communities of Baiao and Oriximina municipalities, Para State, Amazon region, Brazil.

Years	Quilombo remnant communities in Baiao (n=14)				Quilombo remnant communities in Oriximina (n=18)			
	
	Malaria cases	Malaria cases (range)	API (average ± SD*)	API (range)	Malaria cases	Malaria cases (range)	API (mean ± SD)	API (range)
2005	108	0 – 55	28.8 ± 59.7	0 – 189	212	0 – 137	104.7 ± 284.2	0 – 1,104.2
2006	60	0 – 38	14.3 ± 34.6	0 – 130.6	126	0 – 116	35.2 ± 117.1	0 – 489.4
2007	19	0 – 16	4.0 ± 14.7	0 – 55	291	0 – 225	100.5 ± 259.9	0 – 929.4
2008	8	0 – 5	1.1 ± 2.9	0 – 10.3	463	0 – 265	226.1 ± 415.8	0 – 1,450
2009	8	0 – 7	2.0 ± 6.4	0 – 24.1	1,779	0 – 543	665.0 ± 669.7	0 – 2,041.4
2010	41	0 – 16	17.6 ± 38.4	0 – 142.9	167	0 – 69	58.3 ± 84.1	0 – 291.1
2011	156	0 – 46	35.3 ± 43.3	0 – 130.3	22	0 – 19	6.2 ± 19.1	0 – 80.2
2012	59	0 – 16	10.0 ± 12.9	0 – 41.9	1	0 – 1	0.2 ± 0.9	0 – 4.21
2013	3	0 – 1	0.8 ± 1.8	0 – 6	6	0 – 3	3.0 ± 6.4	0 – 20.8
2014	0	0 – 0	0 ± 0	0 – 0	0	0 – 0	0 ± 0	0 – 0
2015	0	0 – 0	0 ± 0	0 – 0	0	0 – 0	0 ± 0	0 – 0
2016	2	0 – 2	0.5 ± 1.8	0 – 6.9	0	0 – 0	0 ± 0	0 – 0
2017	28	0 – 9	4.8 ± 4.7	0 – 13.7	2	0 – 2	0.4 ± 1.8	0 – 7.5
2018	246	0 – 66	53.0 ± 49.2	0 – 174.7	52	0 – 46	11.6 ± 40.7	0 – 172.9
2019	94	0 – 25	21.9 ± 20.2	0 – 70.8	80	0 – 79	9. 6 ± 39.7	0 – 168.8
2020	24	0 – 7	5.6 ± 8.8	0 – 30.1	413	0 – 154	137.8 ± 269.6	0 – 974.2
Total	856	0 – 66	12.5 ± 29.9	0 – 189	3,614	0 – 543	84.9 ± 279.0	0 – 2,041.4

*SD = Standard Deviation.

In Baiao municipality, no recorded cases of malaria infection were found for 2014 and 2015, whereas the remaining years included two (2016) to 246 (2018) cases. In Oriximina, no recorded cases were found for 2014, 2015, and 2016, whereas the remaining years registered from one (2012) to 1,779 (2009) cases. From 2005 to 2020, all 32 localities registered at least one malaria case.

Next, we analyzed API and quantitative data for each of the 32 communities, 14 in Baiao municipality (Table 3) and 18 in Oriximina municipality (Table 4). Based on these data, we selected the five communities with the highest number of cases and their respective years. Among the communities of Baiao municipality were Umarizal (66 cases, 2018), Santa Fe (55 cases, 2005), Baixinha (46 cases, 2011), Joana Peres (44 cases, 2005), and Bailique da Beira (40 cases, 2018). In Oriximina municipality, the highest number of cases was recorded in 2009, in Tapagem (543 cases), Boa Vista (414 cases), Terra Preta II (216 cases), and Moura (168 cases). Still regarding Oriximina municipality, in 2020, the fifth highest number of recorded cases occurred in Parana do Abui, where 154 cases were reported. For this same location, no cases were recorded from 2011 to 2017 confirming the unstable transmission pattern.

**Table 3:** - Annual record of malaria cases and Annual Parasitic Incidence (API) from 2005 to 2020 in each quilombo remnant communities in Baiao municipality, Para State, Amazon region, Brazil.

Quilombo remnant communities in Baiao municipality	Temporal distribution of malaria cases (n) and Annual Parasitic Incidence (API) by year															

	2005		2006		2007		2008		2009		2010		2011		2012	

	n	API	n	API	n	API	n	API	n	API	n	API	n	API	n	API
Araquembaua	0	0	0	0	0	0	0	0	0	0	2	2.9	32	46.1	16	23.1
Baixinha	1	2.8	1	2.8	0	0	0	0	0	0	16	45.3	46	130.3	9	25.5
Bailique da Beira	1	4.4	2	8.7	1	0	0	0	1	4.4	5	21.8	16	69.9	2	8.7
Boa Vista	2	9.9	0	0	0	0	0	0	0	0	0	0	2	9.9	0	0
Calados	0	0	0	0	0	0	0	0	0	0	0	0	0	0	1	1.8
Campelo	2	12	0	0	0	0	0	0	0	0	0	0	16	96.4	3	18.1
Igarapezinho	0	0	0	0	0	0	0	0	0	0	0	0	0	0	0	0
Joana Peres	44	40	13	11.8	2	1.8	5	4.5	0	0	1	0.9	5	4.5	3	2.7
Pampelonia	0	0	1	6	0	0	0	0	0	0	0	0	14	84.3	0	0
Parita Miri	0	0	0	0	0	0	0	0	0	0	0	0	0	0	0	0
Santo Antonio	1	142.9	1	33.3	0	0	0	0	0	0	1	142.9	0	0	0	0
Santa Fe	55	189	38	130.6	16	55	3	10.3	7	24.1	1	3.4	3	10.3	4	13.7
Umarizal	2	1.9	2	1.9	0	0	0	0	0	0	6	5.6	9	8.4	5	4.7
Varginha	0	0	2	5.2	0	0	0	0	0	0	9	23.6	13	34	16	41.9
Total	108	-	60	-	19	-	8	-	8	-	41	-	156	-	59	-
Quilombo remnant communities in Baiao municipality	Temporal distribution of malaria cases (n) and Annual Parasitic Index (API) by year															
	2013		2014		2015		2016		2017		2018		2019		2020	
	n	API	n	API	n	API	n	API	n	API	n	API	n	API	n	API
Araquembaua	1	1.4	0	0	0	0	0	0	2	2.9	33	47.6	17	24.5	5	7.2
Baixinha	0	0	0	0	0	0	0	0	4	11.3	36	102	25	70.8	1	2.8
Bailique da Beira	0	0	0	0	0	0	0	0	2	8.7	40	174.7	11	48	4	17.5
Boa Vista	0	0	0	0	0	0	0	0	1	5	14	89.3	2	9.9	0	0
Calados	0	0	0	0	0	0	0	0	0	0	0	0	0	0	0	0
Campelo	0	0	0	0	0	0	0	0	1	6	11	66.3	7	42.2	0	0
Igarapezinho	0	0	0	0	0	0	0	0	2	8.3	1	4.1	6	24.8	1	4.1
Joana Peres	0	0	0	0	0	0	0	0	9	8.2	12	10.9	5	4.5	0	0
Pampelonia	1	6	0	0	0	0	0	0	0	0	13	78.3	2	12	5	30.1
Parita Miri	0	0	0	0	0	0	0	0	0	0	6	63.2	1	10.5	1	10.5
Santo Antonio	0	0	0	0	0	0	0	0	0	0	1	10.2	1	33.3	0	0
Santa Fe	1	3.4	0	0	0	0	2	6.9	4	13.7	0	0	2	6.9	0	0
Umarizal	0	0	0	0	0	0	0	0	3	2.8	66	61.9	12	11.2	7	6.6
Varginha	0	0	0	0	0	0	0	0	0	0	13	34	3	7.9	0	0
Total	3	-	0	-	0	-	2	-	28	-	246	-	94	-	24	-

**Table 4:** - Annual record of malaria cases and Annual Parasitic Incidence (API) from 2005 to 2020 in each quilombo remnant communities in Oriximina municipality, Para State, Amazon region, Brazil.

Quilombo remnant communities in Oriximina municipality	Temporal distribution of malaria cases (n) and Annual Parasitic Incidence (API) by year															

	2005		2006		2007		2008		2009		2010		2011		2012	

	n	API	n	API	n	API	n	API	n	API	n	API	n	API	n	API
Agua Fria	0	0	0	0	0	0	0	0	82	473.9	8	46.24	0	0	0	0
Araca	53	1,104.2	6	125	31	645.8	25	520.8	31	645.8	2	41.7	1	20.8	0	0
Aracuan	1	6.25	0	0	0	0	0	0	2	8.33	1	6.25	0	0	0	0
Bacabal	0	0	0	0	0	0	1	15.2	3	45.5	0	0	0	0	0	0
Boa Vista	1	4.2	3	12.7	7	29.7	3	12.7	414	1,754.2	16	67.8	0	0	0	0
Cachoeira Porteira	1	2.1	0	0	2	4.3	14	29.9	5	10.7	1	2.1	0	0	0	0
Curuca	0	0	0	0	0	0	12	272.7	45	1,022.7	1	22.7	0	0	0	0
Espirito Santo	137	578	116	489.4	225	949.4	265	1,118	77	324.9	69	291.1	19	80.2	1	4.2
Jamari	3	78.9	0	0	0	0	0	0	34	894.7	1	26.3	0	0	0	0
Jarauaca	1	6.1	0	0	9	54.5	2	12.1	0	0	0	0	0	0	0	0
Jauari	15	105.6	1	7	12	84.5	21	147.9	2	14.1	3	21.1	0	0	0	0
Juquirizinho	0	0	0	0	0	0	29	1,450	34	1,700	5	250	0	0	0	0
Moura	0	0	0	0	0	0	1	5.2	168	866	6	30.9	1	5.2	0	0
Nova Esperanca	0	0	0	0	0	0	0	0	1	166.7	0	0	0	0	0	0
Parana do Abui	0	0	0	0	4	25.3	57	360.8	121	765.8	8	50.6	0	0	0	0
Serrinha	0	0	0	0	1	15.2	0	0	1	15.2	0	0	0	0	0	0
Tapagem	0	0	0	0	0	0	18	67.7	543	2,041.4	34	127.8	0	0	0	0
Terra Preta II	0	0	0	0	0	0	6	57.1	216	1,220.3	12	64.5	1	5.4	0	0
Total	212	-	126	-	291	-	454	-	1.779	-	167	-	22	-	1	-
Quilombo remnant communities in Oriximina municipality	Temporal distribution of malaria cases (n) and Annual Parasitic Incidence (API) by year															
	2013		2014		2015		2016		2017		2018		2019		2020	
	n	API	n	API	n	API	n	API	n	API	n	API	n	API	n	API
Agua Fria	0	0	0	0	0	0	0	0	0	0	0	0	0	0	4	23.1
Araca	1	20.8	0	0	0	0	0	0	0	0	0	0	0	0	0	0
Aracuan	0	0	0	0	0	0	0	0	0	0	0	0	0	0	0	0
Bacabal	1	15.2	0	0	0	0	0	0	0	0	0	0	0	0	0	0
Boa Vista	0	0	0	0	0	0	0	0	0	0	0	0	0	0	5	21.2
Cachoeira Porteira	0	0	0	0	0	0	0	0	0	0	0	0	79	168.8	109	232.9
Curuca	0	0	0	0	0	0	0	0	0	0	0	0	0	0	1	22.9
Espirito Santo	3	12.6	0	0	0	0	0	0	0	0	0	0	0	0	0	0
Jamari	0	0	0	0	0	0	0	0	0	0	0	0	0	0	0	0
Jarauaca	0	0	0	0	0	0	0	0	0	0	0	0	0	0	0	0
Jauari	0	0	0	0	0	0	0	0	0	0	0	0	0	0	0	0
Juquirizinho	0	0	0	0	0	0	0	0	0	0	0	0	0	0	13	650
Moura	0	0	0	0	0	0	0	0	0	0	0	0	0	0	2	10.3
Nova Esperanca	0	0	0	0	0	0	0	0	0	0	0	0	0	0	0	0
Parana do Abui	0	0	0	0	0	0	0	0	0	0	4	25.3	0	0	154	974.2
Serrinha	0	0	0	0	0	0	0	0	0	0	0	0	0	0	0	0
Tapagem	0	0	0	0	0	0	0	0	2	7.5	46	172.9	1	3.8	78	293.2
Terra Preta II	1	5.4	0	0	0	0	0	0	0	0	2	10.7	0	0	47	252.7
Total	6	-	0	-	0	-	0	-	2	-	52	-	80	-	413	-

When analyzing the cases by infecting species, *P. vivax* was the most prevalent in all communities and throughout the entire period, namely 91.2% (781/856) in Baiao municipality communities and 72.5% (2,622/3,614) in Oriximina municipality communities (Table 5). *P. falciparum* infections were detected in two areas, recording many cases in Oriximina municipality communities, 25.5% (923/3,614). This is a remarkable finding, given that *P. falciparum* infections are responsible for the most severe clinical cases of the disease.

**Table 5:** - Malaria cases by *Plasmodium* spp. over 16 years in 32 quilombo remnant communities of Baiao and Oriximina municipalities, Para State, Amazon region, Brazil.

Years	Quilombo remnant communities in Baiao municipality (n=14)							Quilombo remnant communities in Oriximina municipality (n=18)						

	*P. vivax*		*P. falciparum*			Others		*P. vivax*		*P. falciparum*			Others	

	n	%	n		%	n	%	n	%	n		%	n	%
2005	78	72.2	28		25.9	2	1.9	210	99.0	1		0.5	1	0.5
2006	53	88.3	6		10	1	1.7	126	100	0		0	0	0
2007	16	84.2	2		10.5	1	5.3	180	61.8	107		36.8	4	1.4
2008	6	75	1		12.5	1	12.5	348	75.2	109		23.5	6	1.3
2009	8	100	0		0	0	0	1,032	58.0	691		38.8	56	3.1
2010	41	100	0		0	0	0	151	90.4	14		8.4	2	1.2
2011	155	99.3	1		0.6	0	0	22	100	0		0	0	0
2012	58	98.3	1		1.7	0	0	1	100	0		0	0	0
2013	1	33.3	2		66.7	0	0	6	100	0		0	0	0
2014	0	0	0		0	0	0	0	0	0		0	0	0
2015	0	0	0		0	0	0	0	0	0		0	0	0
2016	2	100	0		0	0	0	0	0	0		0	0	0
2017	22	78.6	0		0	6	21.4	2	100	0		0	0	0
2018	227	92.3	0		0	19	7.7	52	100	0		0	0	0
2019	90	95.7	0		0	4	4.3	79	98.7	1		1.2	0	0
2020	24	100	0		0	0	0	413	100	0		0	0	0
Total	781	91.2	41		4.8	34	4.0	2,622	72.5	923		25.5	69	2.0

n = total number of malaria cases by year. % = percentage.

## DISCUSSION

Para State is among the five states with the most quilombo remnant communities in Brazil^10^. The entire population living in the Northern region of Brazil, including individuals residing in these communities, is at risk for malaria infection. This risk was calculated based on the annual parasite index (API), in which scores above 50 classifies the area as high risk. Malaria does not occur homogeneously throughout Para State since API indexes have ranged from no risk to high-risk areas over the last two decades^5,7,19^.

The quilombo remnant communities are concentrated in the northernmost and southernmost areas of the Baiao and Oriximina municipalities, respectively. Considering that most cases of malaria occur in rural areas of Para, it is expected that transmission is restricted to a given area and can occur interspersed in localities that are geographically close. However, transmission rates were not continuous, as there was considerable variation between communities in the same municipality, as well as communities without case notification for several periods.

Although the transmission is unstable, it should be noted that all 32 quilombo communities in Baiao and Oriximina municipalities registered at least one case of malaria every year from 2005 and 2020. Other quilombo remnant communities are in the process of certification, indicating that the number of individuals living in quilombo remnant communities is projected to increase over the next few years. Despite quilombo populations being recognized for their value in the cultural formation of Brazil, they are still in a highly vulnerable situation, with little attention given to their needs as citizens, including basic healthcare^8,12,13,20^.

We observed that, although infected individuals continue to circulate among the affected communities, a considerable variability is noted regarding malaria incidence within each community. This may be due to the proximity between these communities and the impact of malaria control actions, which resulted in a focal transmission profile for both areas. This is common in the Brazilian Amazon, where transmission is unstable^7,19,21^. These communities are close to urban areas with easier access to laboratorial diagnosis, which consequently aid on controlling and managing the disease among individuals living in the same community.

Baiao municipality presented a lower number of cases, despite harboring a quilombo remnant population twice the size of that of Oriximina municipality. The total number of residents was estimated to be 5,747 and 2,555 in Baiao and Oriximina municipalities, respectively. These municipalities are located in different parts of Para State, and considering the forest area, Oriximina municipality is covered by 94% of forest, whereas Baiao municipality by only 66%^16^. The higher malaria incidence in Oriximina municipality may be caused by mining activities. Previous studies have shown the impact of mining activities on malaria transmission in the Amazon^5,7^. A study conducted in Oriximina municipality investigated the health conditions of quilombo women living in eight communities located around the banks of the Trombetas river^20^; however, malaria was not reported as a common health problem. This is expected given that men are usually more exposed to the risk of malaria infections in Brazil^18^.

Concerning the parasite species that cause malaria, 76.1% of total cases in all analyzed communities were caused by *P. vivax*. This finding agrees with a previous report on data from all states in the Amazon region spanning 28 years, which showed that 73.5% of malaria cases were caused by *P. vivax* ^14^. Since the 1980s, *P. vivax* has been the most prevalent parasite throughout the Northern region of Brazil and is currently the cause of approximately 85% of malaria cases^4,5,14^.

A relevant contribution of our study was determining that 10.9% and 39.1% of the malaria cases in Baiao and Oriximina municipalities, respectively, occurred in quilombo remnant communities. An important finding is the high percentage of malaria cases caused by *P. falciparum* in the Oriximina municipality quilombo communities (25.5%), whereas Baiao municipality registered only 4.8% during the studied period (2005 to 2020). As *P. falciparum* is responsible for severe clinical cases of this disease, as well as for the high mortality rates of malaria worldwide^3,21^, surveillance actions must be continuous and appropriate for this epidemiological situation, considering that malaria cases were registered based on the place of probable infection, where most individuals are quilombolas.

A limitation of our study is the lack of updated data on the population size of each community, which is used for calculating the API. Over 16 years, we observed that API was calculated several times using the same population size. On the other hand, this number may also represent a close estimate of the real population size, given the low level of migration and the fact that many of these communities are formed by few families^8^. Considering this limitation, we did not initially classify the areas according to risk and opted instead for a more descriptive analysis of the number of cases in certified communities and the total of cases in noncertified communities. For the complete register, we included data on the variability of the annual incidence rates, given that this is the first study on malaria in quilombo remnant populations from the Para State and Amazon region, and this classification of risk areas can help to implement effective measures of control by the SIVEP-Malaria.

In general, previous studies have focused on social, economic, and other health aspects of this population^12,13,20^. Few investigations have shown data on infectious and parasitic diseases in quilombo communities, namely the prevalence of human T-lymphotropic virus (HTLV) infection^22^, the seroprevalence of rodent-borne viruses^23^, the molecular characterization of noroviruses from quilombola children^24^, and the occurrence of intestinal parasites^25,26^.

There are few studies on African descendants, considering malaria and countries in South America. In Brazil, there are data on the molecular analysis of glucose-6-phosphate dehydrogenase (G6PD) mutations and the Duffy blood group in African descendant communities from the Brazilian Amazon. This study showed that 24.3% of people were Duffy-negative and 41.3% were heterozygous for FY*B^ES^. The frequency of allele FY*B^ES^ was 41.0%. The results emphasize the need to monitor G6PD deficiency for the use of primaquine in the routine care of the Afro-descendant communities of the Trombetas river^27^. As the percentage of Duffy-negative was low in some communities included in our study, it can explain, in part, the high prevalence of *P. vivax.* However, for *P. falciparum* cases registered in Oriximina municipality, this may have been a problem in the measures of control, and it is important information for the SIVEP-Malaria. Additionally, 23% of malaria cases reported in an epidemiological study from Colombia were Afro-descendant individuals^28^.

Among factors associated with the lack of adequate access to healthcare, many quilombola individuals reported their place of residency as an important barrier. This association was not found for the Northern region of Brazil but the study did not focus on specific situations, such as access to diagnosis and treatment of malaria^29^. Quilombo communities are generally located in rural areas of Brazil, so they may have very limited access to healthcare services^30^. Therefore, continuously providing access to diagnosis and treatment for infectious and parasitic diseases, such as malaria, in the endemic areas in the Bazilian Amazon basian continues to be crucial for improving health conditions in these communities.

In this study, we characterized the investigated areas regarding malaria cases from almost two decades and included data from the complete SIVEP-Malaria database from the Brazilian Ministry of Health (2003–2020), excluding the first two years to avoid any inconsistency in data recorded. This descriptive and retrospective analysis revealed significant temporal variation in the reported cases, reinforcing that the quilombo population inhabits malaria-endemic areas. In these locations, infection by the *P. vivax* parasite was more prevalent, with focal and unstable transmission and large variation in parasite incidence when comparing communities within the same municipality and between the Baiao and Oriximina municipalities. Therefore, this study demonstrated that quilombo remnant communities in the Amazon region present a similar epidemiological profile of malaria.

We highlight that malaria control has proven to be successful. However, the implementation of effective measures to ensure appropriate early diagnosis and treatments is still necessary. Therefore, the purpose of our study was to improve the understanding on malaria exposure in quilombo communities, given the current lack of data in the literature. We hope to contribute to the improvement of health conditions in this vulnerable population residing in areas at high risk of infectious diseases, as well as all individuals that are living in malaria-endemic areas in the Brazilian Amazon.

## CONCLUSION

This study is the first to describe malaria transmission among people living in quilombo remnant communities in the Brazilian Amazon region. In the study area, over a 16-year period, the quilombo remnant communities residing in the Baiao and Oriximina municipalities registered 10.9% and 39.1%, respectively, of the total malaria cases in each municipality, accounting for 0.44% of the total malaria cases recorded in Para State. These individuals are at risk of malaria, and the variations in the number of cases and annual incidence of infection over time indicate unstable and focal transmission.
